# Supplementary figures and images for: Fungal microbiome in gut of systemic lupus erythematosus (SLE)-prone mice (pristane and FCGRIIb deficiency), a possible impact of fungi in lupus
Source: PLoS One. 2024 Dec 5;19(12):e0314662. doi: 10.1371/journal.pone.0314662 (PMC11620554; doi:10.1371/journal.pone.0314662)

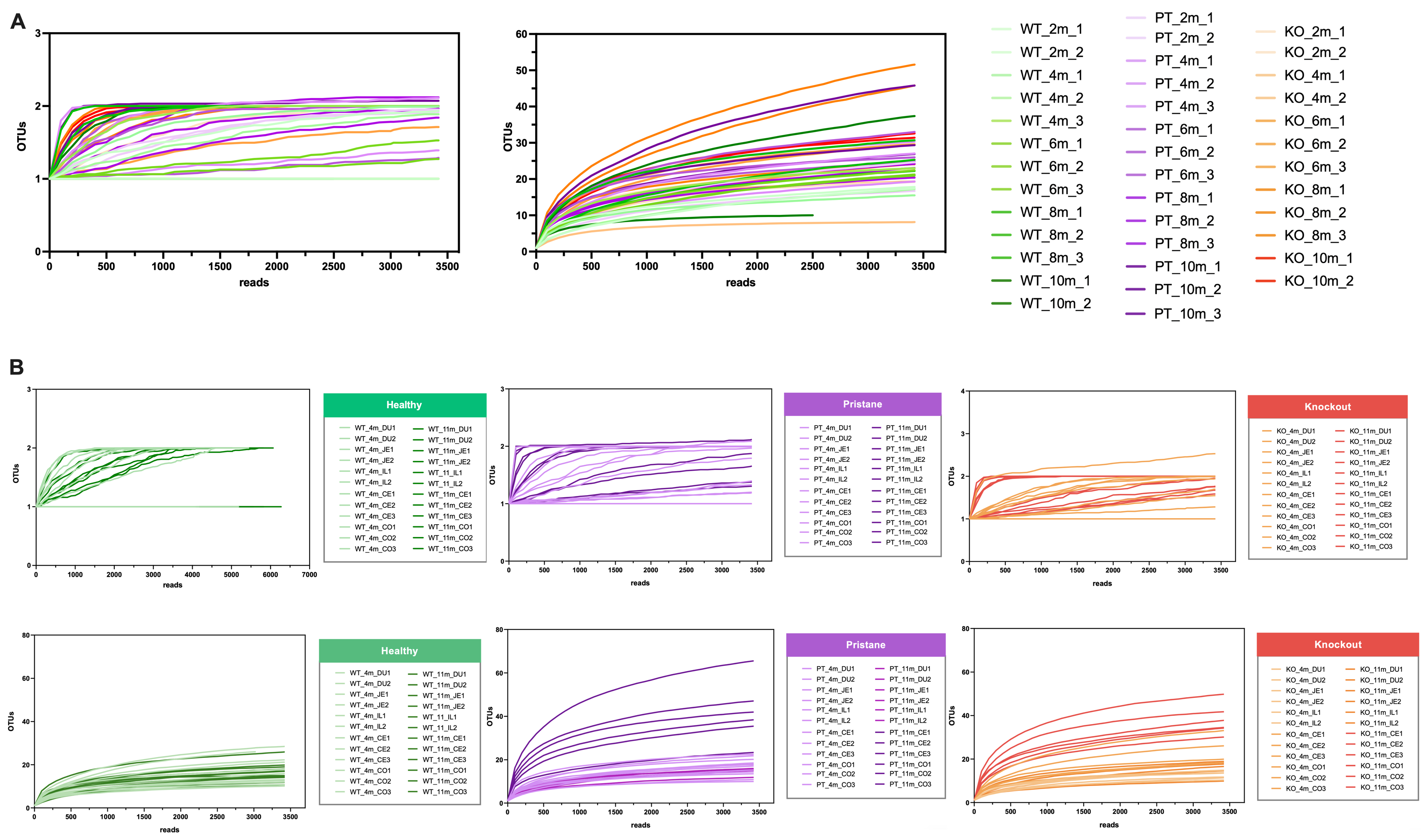

Supplement: S1 Fig — Rarefaction curves of quality reads per sample at phylum and genus operational taxonomic units (OTUs) of (A) all samples, (B) separate groups of healthy, pristane and KO samples. (TIFF) [file pone.0314662.s001.tiff]

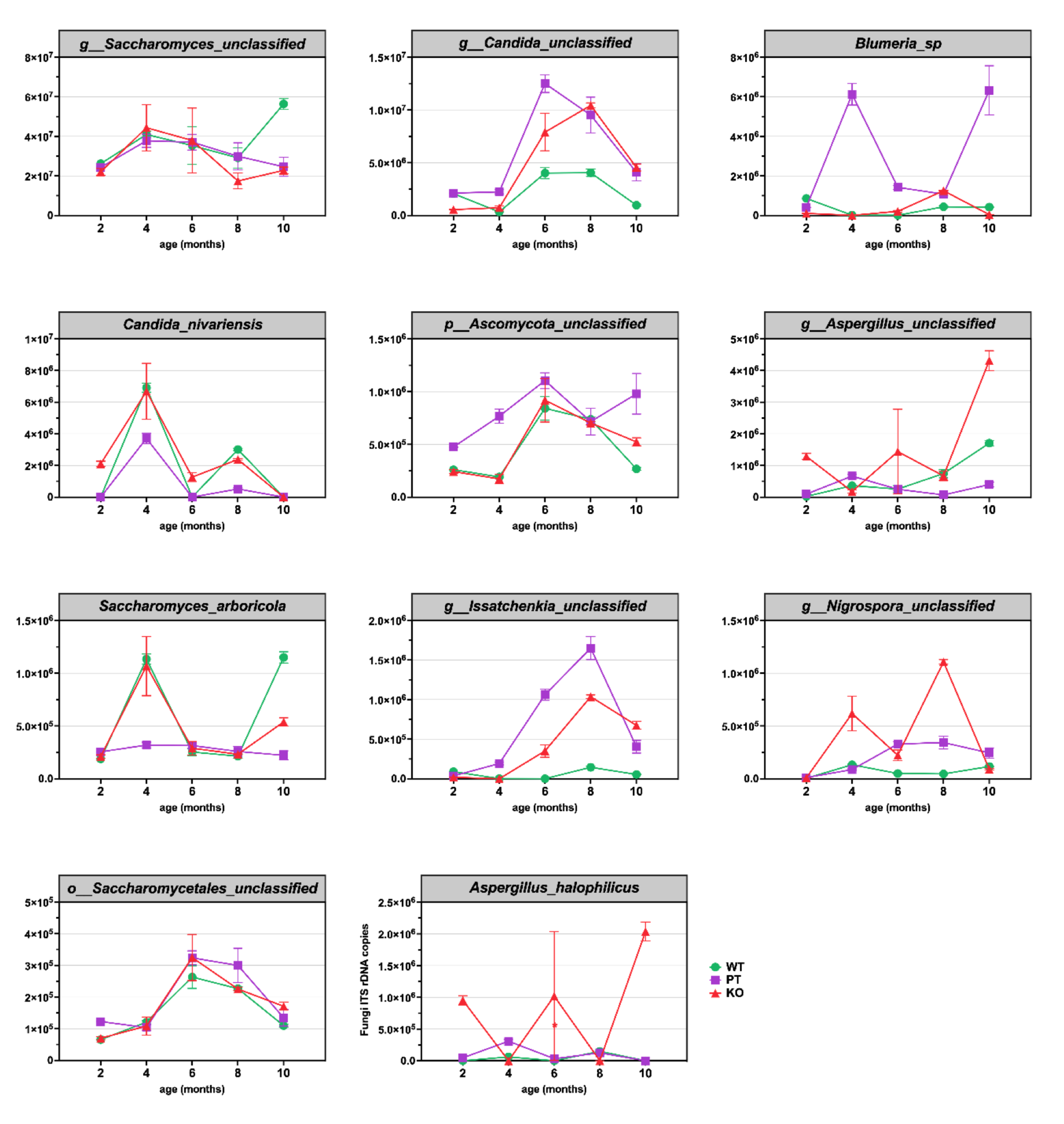

Supplement: S2 Fig — (TIFF) [file pone.0314662.s002.tiff]

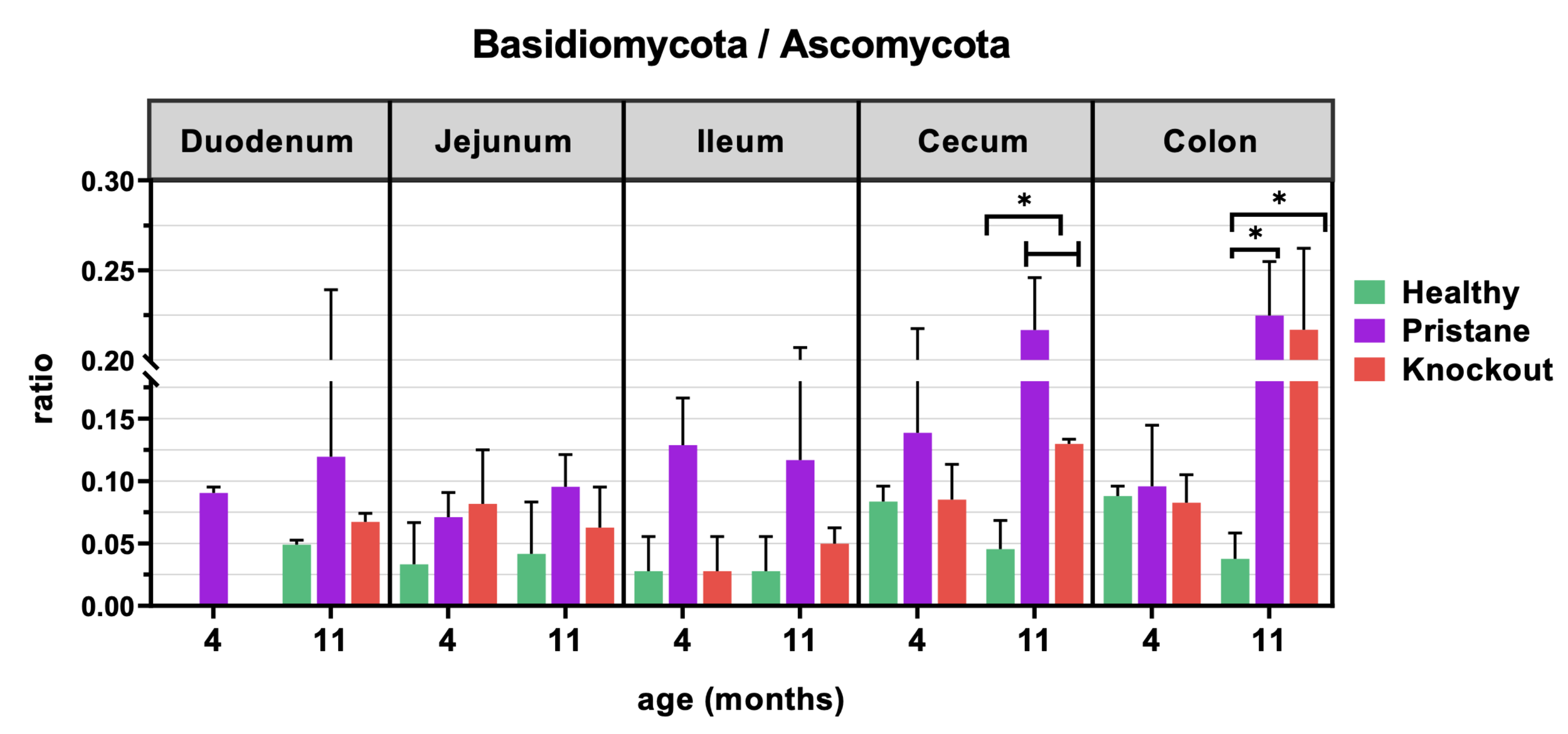

Supplement: S3 Fig — * represents statistically significant ANOVA P < 0.05, in comparison with healthy at 11 months. (TIFF) [file pone.0314662.s003.tiff]

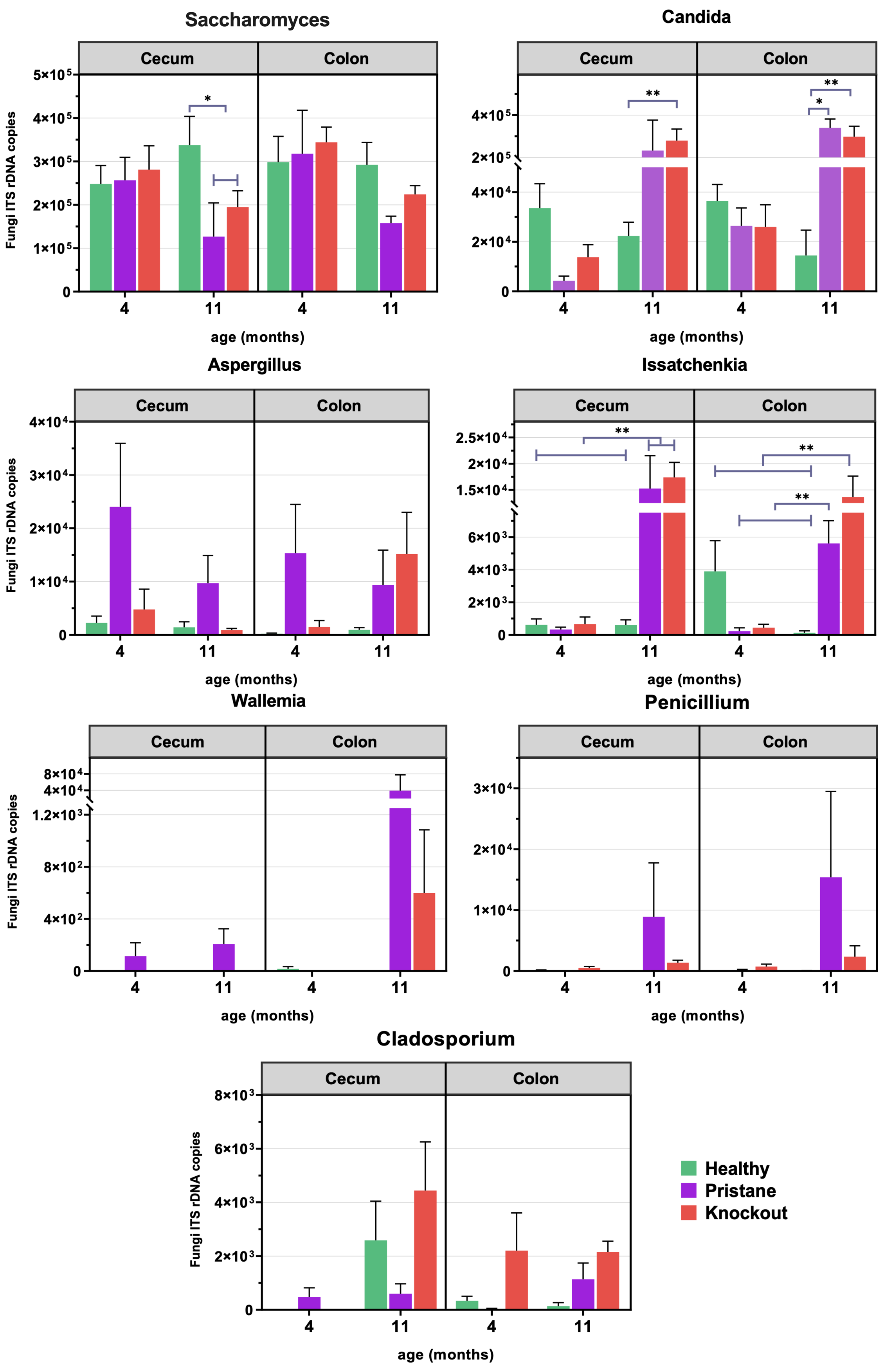

Supplement: S4 Fig — Noted that genera Blumeria, Asomycota, Nigrospora and Fusarium were not displayed as they exhibited no statistical difference. *, ** and *** represent ANOVA P < 0.05, 0.01, and 0.001, respectively. (TIFF) [file pone.0314662.s004.tiff]

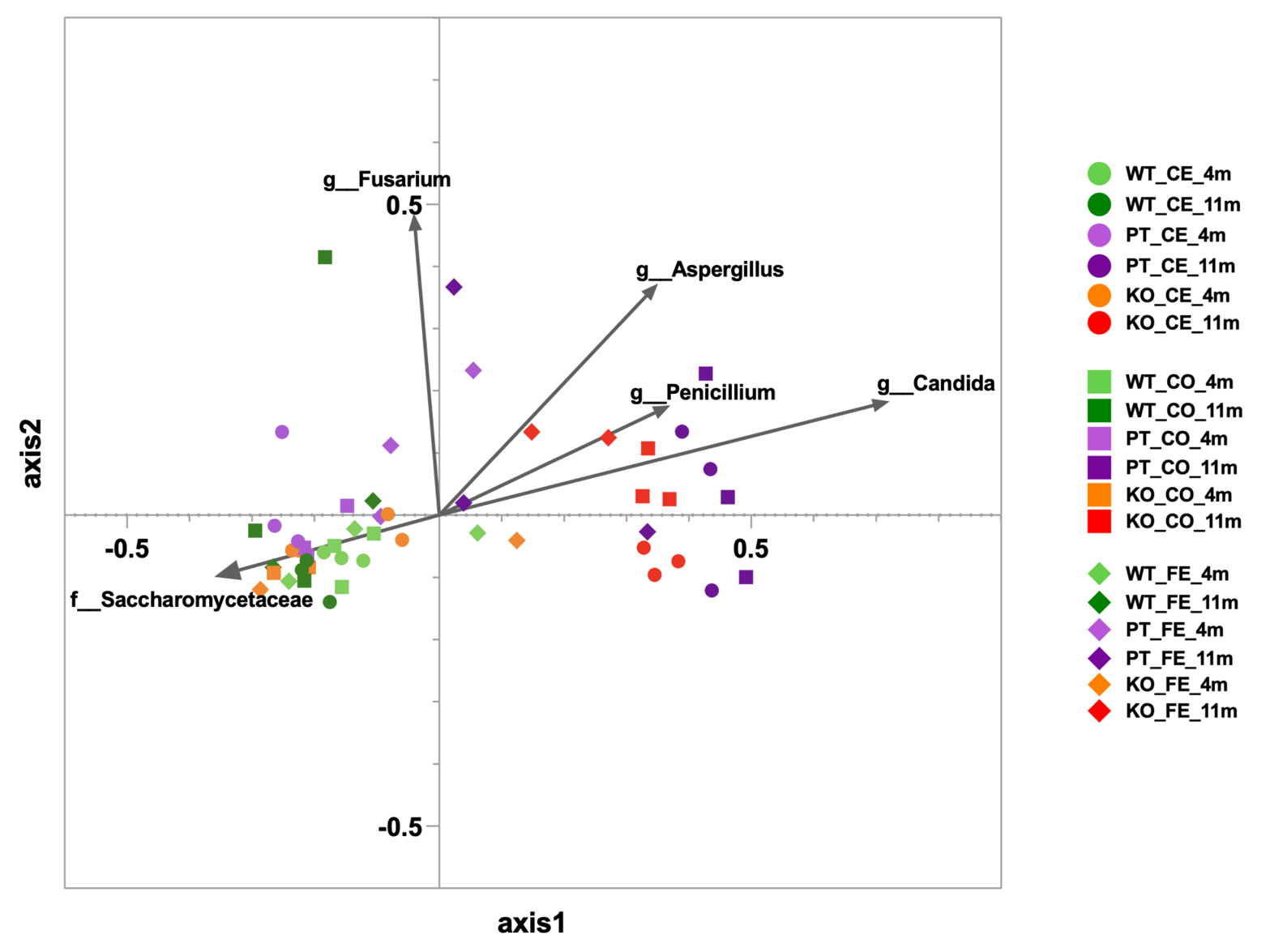

Supplement: S5 Fig — (TIFF) [file pone.0314662.s005.tiff]
