# Supplementary material for: Fungal microbiome in gut of systemic lupus erythematosus (SLE)-prone mice (pristane and FCGRIIb deficiency), a possible impact of fungi in lupus
Source: PLoS One. 2024 Dec 5;19(12):e0314662. doi: 10.1371/journal.pone.0314662 (PMC11620554; doi:10.1371/journal.pone.0314662)
Supplement: S1 Table — Alpha diversity estimates at genus level of wild-type (WT), pristane (PT) and FcgRIIb-/- (KO) mice of (A) feces at 2, 4, 6, 8 and 10 months; and (B) five intestinal parts (duodenum, jejunum, ileum, cecum, and colon) at 4 and 11 months of age. (PDF) [file pone.0314662.s006.pdf]

A

Samples	Good's coverage (%)	OTUs	Alpha diversity	
			Chao	Shannon
WT_2m_1	99.80	17	25.39	0.63
WT_2m_2	99.82	18	28.80	0.87
WT_4m_1	99.88	16	19.61	0.85
WT_4m_2	99.82	17	22.86	0.30
WT_4m_3	99.85	23	27.25	0.82
WT_6m_1	99.79	21	27.08	0.88
WT_6m_2	99.79	22	29.61	0.93
WT_6m_3	99.79	21	28.29	0.91
WT_8m_1	99.82	22	28.57	0.93
WT_8m_2	99.74	25	34.02	0.94
WT_8m_3	99.81	31	36.51	1.23
WT_10m_1	99.58	37	53.04	0.98
WT_10m_2	99.96	15	15.00	0.52

Samples	Good's coverage (%)	OTUs	Alpha diversity	
			Chao	Shannon
PT_2m_2	99.75	17	33.11	0.94
PT_4m_1	99.84	25	24.17	1.02
PT_4m_2	99.76	19	36.27	0.88
PT_4m_3	99.83	27	34.61	1.40
PT_6m_1	99.87	31	29.78	1.34
PT_6m_2	99.84	26	30.36	1.09
PT_6m_3	99.73	25	42.68	1.17
PT_8m_1	99.79	33	31.07	1.14
PT_8m_2	99.85	24	24.66	1.07
PT_8m_3	99.79	20	32.15	0.95
PT_10m_1	99.56	23	61.57	1.20
PT_10m_2	99.81	45	36.16	1.32
PT_10m_3	99.79	29	34.01	1.50

Samples	Good's coverage (%)	OTUs	Alpha diversity	
			Chao	Shannon
KO_2m_1	99.85	23	27.78	1.24
KO_2m_2	99.92	19	20.27	0.58
KO_4m_1	99.97	8	8.37	0.14
KO_4m_2	99.91	19	21.37	1.07
KO_6m_1	99.81	30	35.46	1.15
KO_6m_2	99.78	29	35.92	1.04
KO_6m_3	99.75	25	33.21	0.88
KO_8m_1	99.40	51	79.19	1.36
KO_8m_2	99.45	46	70.18	1.39
KO_8m_3	99.84	24	29.20	1.34
KO_10m_1	99.82	31	35.48	1.45
KO_10m_2	99.73	33	40.37	1.32

B

Samples	Good's coverage (%)	OTUs	Alpha diversity	
			Chao	Shannon
WT_4m_DU1	99.94	10	11.20	0.17
WT_4m_DU2	99.94	13	13.40	0.22
WT_4m_JE1	99.84	16	22.30	0.31
WT_4m_JE2	99.94	10	10.98	0.15
WT_4m_IL1	99.91	13	15.34	0.22
WT_4m_IL2	99.97	11	11.05	0.14
WT_4m_CE1	99.70	25	29.47	0.62
WT_4m_CE2	99.84	21	24.03	0.59
WT_4m_CE3	99.94	12	12.53	0.58
WT_4m_CO1	99.86	22	25.30	0.64
WT_4m_CO2	99.83	28	30.59	0.82
WT_4m_CO3	99.86	18	22.48	0.72
WT_11m_DU1	99.91	12	13.60	0.29
WT_11m_DU2	99.90	14	15.92	0.24
WT_11m_JE1	99.84	17	24.74	0.35
WT_11m_JE2	99.93	14	15.32	0.35
WT_11_IL1	99.93	11	12.24	0.13
WT_11_IL2	99.91	15	16.50	0.30

WT_11m_CE1	99.81	26	33.11	0.79
WT_11m_CE2	99.86	20	25.20	0.47
WT_11m_CE3	99.87	17	20.23	0.48
WT_11m_CO1	99.89	19	21.25	0.45
WT_11m_CO2	99.96	15	15.50	1.05
WT_11m_CO3	99.85	18	23.00	0.63

**Samples**	**Good's coverage (%)**	**OTUs**	**Alpha diversity**	
			**Chao**	**Shannon**
PT_4m_DU1	99.92	11	12.99	0.15
PT_4m_DU2	99.95	10	10.76	0.16
PT_4m_JE1	99.90	14	16.54	0.45
PT_4m_JE2	99.87	15	20.86	0.32
PT_4m_IL1	99.93	14	14.81	0.81
PT_4m_IL2	99.88	18	21.99	0.73
PT_4m_CE1	99.85	18	22.16	0.53
PT_4m_CE2	99.88	20	23.03	0.54
PT_4m_CE3	99.87	22	26.04	1.02
PT_4m_CO1	99.84	18	24.01	0.54
PT_4m_CO2	99.86	17	21.94	0.51
PT_4m_CO3	99.83	22	28.04	0.78
PT_11m_DU1	99.89	15	18.69	0.23
PT_11m_DU2	99.92	12	13.72	0.24
PT_11m_JE1	99.85	16	19.69	0.26
PT_11m_JE2	99.89	16	18.57	0.41
PT_11m_IL1	99.86	15	20.09	0.47
PT_11m_IL2	99.88	16	19.83	0.74
PT_11m_CE1	99.76	42	49.65	1.73
PT_11m_CE2	99.70	47	56.53	1.73
PT_11m_CE3	99.72	35	46.73	1.34
PT_11m_CO1	99.55	65	80.25	1.89
PT_11m_CO2	99.72	38	48.22	1.31
PT_11m_CO3	99.79	23	32.36	1.51

**Samples**	**Good's coverage (%)**	**OTUs**	**Alpha diversity**	
			**Chao**	**Shannon**
KO_4m_DU1	99.88	14	17.40	0.14
KO_4m_DU2	99.94	10	11.52	0.15
KO_4m_JE1	99.93	12	12.93	0.15
KO_4m_JE2	99.91	14	15.64	0.26
KO_4m_IL1	99.87	12	15.95	0.15
KO_4m_IL2	99.86	15	24.16	0.26
KO_4m_CE1	99.81	18	28.61	0.53

KO_4m_CE2	99.89	15	17.92	0.34
KO_4m_CE3	99.84	19	24.47	0.47
KO_4m_CO1	99.70	33	43.84	0.88
KO_4m_CO2	99.88	20	23.40	0.59
KO_4m_CO3	99.81	26	30.65	0.89
KO_11m_DU1	99.83	18	24.09	0.31
KO_11m_DU2	99.80	17	24.46	0.23
KO_11m_JE1	99.85	14	18.85	0.22
KO_11m_JE2	99.86	19	24.31	0.40
KO_11m_IL1	99.88	15	18.34	0.33
KO_11m_IL2	99.92	10	11.71	0.17
KO_11m_CE1	99.71	34	45.23	1.27
KO_11m_CE2	99.75	35	42.81	1.55
KO_11m_CE3	99.80	30	35.87	1.23
KO_11m_CO1	99.69	38	51.12	1.46
KO_11m_CO2	99.70	50	57.62	1.81
KO_11m_CO3	99.80	42	46.05	1.51
