# Supplementary material for: Fungal microbiome in gut of systemic lupus erythematosus (SLE)-prone mice (pristane and FCGRIIb deficiency), a possible impact of fungi in lupus
Source: PLoS One. 2024 Dec 5;19(12):e0314662. doi: 10.1371/journal.pone.0314662 (PMC11620554; doi:10.1371/journal.pone.0314662)
Supplement: Attachment — Submitted filename: Response to Reviewers.docx [file pone.0314662.s007.docx]

Decision: Revision required [PONE-D-24-20227R1] - [EMID:2e0c38865e667e61]

Dear editor and reviewers,

We revised and corrected the manuscripts (using track changes) following the comments. **Line numbers are based on the "Manuscript w/ Track Changes (Simple Markup)" file.**

Sincerely yours,

Naraporn Somboonna (corresponding author)

Asada Leelahavanichkul (co-corresponding author)

**Editor comments (**Veena Taneja**)**

Thank you for submitting your manuscript to PLOS ONE. After careful consideration, we feel that it has merit but does not fully meet PLOS ONE’s publication criteria as it currently stands. Therefore, we invite you to submit a revised version of the manuscript that addresses the points raised during the review process.
==============================
**ACADEMIC EDITOR:**

**The manuscript has certain statements that need clarification. To make it suitable for all readers, please make sure to read it for proper English so it is easily understood.**

**Response:** Clarified the certain statements, and made sure for proper English.
==============================

Please submit your revised manuscript by Dec 23 2024 11:59PM. If you will need more time than this to complete your revisions, please reply to this message or contact the journal office at [plosone@plos.org](mailto:plosone@plos.org). When you're ready to submit your revision, log on to https://www.editorialmanager.com/pone/ and select the 'Submissions Needing Revision' folder to locate your manuscript file.

Journal Requirements:
Please review your reference list to ensure that it is complete and correct. If you have cited papers that have been retracted, please include the rationale for doing so in the manuscript text, or remove these references and replace them with relevant current references. Any changes to the reference list should be mentioned in the rebuttal letter that accompanies your revised manuscript. If you need to cite a retracted article, indicate the article’s retracted status in the References list and also include a citation and full reference for the retraction notice.

**Response:** Ensured reference list is complete and correct.

Additional Editor Comments:
Please revise the article for proper English.
Line 145--- :At complete study, mice----"
Line 159 " what does it mean " All mice were measured SLE characteristics"
**Response:** Revised L145 and clarified L159.
Clarify these statements and proof for other mistakes.
**Response:** Proofread English. Thank you very much.

Reviewers' comments:
Reviewer's Responses to Questions

**Comments to the Author**

1. If the authors have adequately addressed your comments raised in a previous round of review and you feel that this manuscript is now acceptable for publication, you may indicate that here to bypass the “Comments to the Author” section, enter your conflict of interest statement in the “Confidential to Editor” section, and submit your "Accept" recommendation.

Reviewer #1: (No Response)

Reviewer #3: All comments have been addressed

2. Is the manuscript technically sound, and do the data support the conclusions?
The manuscript must describe a technically sound piece of scientific research with data that supports the conclusions. Experiments must have been conducted rigorously, with appropriate controls, replication, and sample sizes. The conclusions must be drawn appropriately based on the data presented.

Reviewer #1: (No Response)

Reviewer #3: Yes

3. Has the statistical analysis been performed appropriately and rigorously?

Reviewer #1: (No Response)

Reviewer #3: N/A

4. Have the authors made all data underlying the findings in their manuscript fully available?
The [PLOS Data policy](http://track.editorialmanager.com/CL0/http:%2F%2Fwww.plosone.org%2Fstatic%2Fpolicies.action%23sharing/1/010f01930c225f4a-91bbfd11-7c70-4fc0-9524-a53b74adeca6-000000/RGDyDBb4ZhV3lNIbconZAp9bsvC5prTsUISnbgl6kWw=184) requires authors to make all data underlying the findings described in their manuscript fully available without restriction, with rare exception (please refer to the Data Availability Statement in the manuscript PDF file). The data should be provided as part of the manuscript or its supporting information, or deposited to a public repository. For example, in addition to summary statistics, the data points behind means, medians and variance measures should be available. If there are restrictions on publicly sharing data—e.g. participant privacy or use of data from a third party—those must be specified.

Reviewer #1: (No Response)

Reviewer #3: Yes

5. Is the manuscript presented in an intelligible fashion and written in standard English?
PLOS ONE does not copyedit accepted manuscripts, so the language in submitted articles must be clear, correct, and unambiguous. Any typographical or grammatical errors should be corrected at revision, so please note any specific errors here.

Reviewer #1: (No Response)

Reviewer #3: Yes

6. Review Comments to the Author
Please use the space provided to explain your answers to the questions above. You may also include additional comments for the author, including concerns about dual publication, research ethics, or publication ethics. (Please upload your review as an attachment if it exceeds 20,000 characters)

Reviewer #1: (No Response)

Reviewer #3: Though this study is not anew subject, it has great impact on understanding how autoimmune diseases operate and has impact in translational medicine and inventing new immunological treatments

7. PLOS authors have the option to publish the peer review history of their article ([what does this mean?](https://track.editorialmanager.com/CL0/https:%2F%2Fjournals.plos.org%2Fplosone%2Fs%2Feditorial-and-peer-review-process%23loc-peer-review-history/1/010f01930c225f4a-91bbfd11-7c70-4fc0-9524-a53b74adeca6-000000/39ayEHKncGv2KRH5-l_t0JcesvYLfafpkz0vO2vZeS0=184)). If published, this will include your full peer review and any attached files.
If you choose “no”, your identity will remain anonymous but your review may still be made public.

**Do you want your identity to be public for this peer review?** For information about this choice, including consent withdrawal, please see our [Privacy Policy](https://track.editorialmanager.com/CL0/https:%2F%2Fwww.plos.org%2Fprivacy-policy/1/010f01930c225f4a-91bbfd11-7c70-4fc0-9524-a53b74adeca6-000000/M1a93Hkmlxe2-jwXyvCNFijFPcMDURPXmkgAG-OSPaQ=184).

Reviewer #1: No

Reviewer #3: **Yes:** Becky Abdissa Adugna

2. Has the statistical analysis been performed appropriately and rigorously?

Reviewer #1: Yes

Reviewer #2: Yes
