# Supplementary material for: Fungal microbiome in gut of systemic lupus erythematosus (SLE)-prone mice (pristane and FCGRIIb deficiency), a possible impact of fungi in lupus
Source: PLoS One. 2024 Dec 5;19(12):e0314662. doi: 10.1371/journal.pone.0314662 (PMC11620554; doi:10.1371/journal.pone.0314662)
Supplement: Attachment — Submitted filename: Response to Reviewers.docx [file pone.0314662.s008.docx]

Decision: Revision required [PONE-D-24-20227R2] - [EMID:45c95f34cb950ce7]

**Editor comments (**Veena Taneja**)**

Journal Requirements:
Please review your reference list to ensure that it is complete and correct. If you have cited papers that have been retracted, please include the rationale for doing so in the manuscript text, or remove these references and replace them with relevant current references. Any changes to the reference list should be mentioned in the rebuttal letter that accompanies your revised manuscript. If you need to cite a retracted article, indicate the article’s retracted status in the References list and also include a citation and full reference for the retraction notice.

**Response:** Added reference, and ensured reference list is complete and correct.

Additional Editor Comments:
Revised sentences are not grammatically correct.
Please get the manuscript checked for English.
**Response:** Revised grammar and checked English.

Thank you very much.

Sincerely yours,

Naraporn Somboonna
